# Consequences of gestational diabetes mellitus on neonatal cardiovascular health: MySweetHeart Cohort study

**DOI:** 10.1038/s41390-022-02390-4

**Published:** 2022-11-28

**Authors:** Stefano C. Di Bernardo, Sebastiano A. G. Lava, Adina Mihaela Epure, Sandrine Estoppey Younes, Arnaud Chiolero, Nicole Sekarski, on behalf of MySweetHeart Research Group, Amar Arhab, Pascal Bovet, Arnaud Chiolero, Stefano Di Bernardo, Adina Mihaela Epure, Leah Gilbert, Justine Gross, Antje Horsch, Stefano Lanzi, Seyda Mayerat, Yvan Mivelaz, Jardena J. Puder, Dan Quansah, Jean‐Benoit Rossel, Umberto Simeoni, Bobby Stuijfzand, Yvan Vial

**Affiliations:** 1 Pediatric Cardiology, Women-Mother-Child Department, Lausanne University Hospital, Lausanne, Switzerland; 2 Heart Failure and Transplantation, Department of Paediatric Cardiology, Great Ormond Street Hospital, London, UK; 3 Population Health Laboratory (#PopHealthLab), University of Fribourg, Fribourg, Switzerland; 4 Department of Epidemiology and Health Services, Center for Primary Care and Public Health (Unisanté), University of Lausanne, Lausanne, Switzerland; 5 Department of Epidemiology, Biostatistics, Occupational Health, School of Population and Global Health, McGill University, Montreal, QC Canada; 6 Institute of Primary Health Care (BIHAM), University of Bern, Bern, Switzerland; 7 Gynecology and Obstetrics, Women, Mother, Child Department, Lausanne University Hospital, Lausanne, Switzerland; 8 Institution of Social and Preventive Medicine, Lausanne University Hospital, Lausanne, Switzerland; 9 Endocrinology, Diabetes and Metabolism, Gynecology and Obstetrics, Lausanne University Hospital, Lausanne, Switzerland; 10 Institute of Higher Education and Research in Healthcare, University of Lausanne, Lausanne, Switzerland; 11 Angiology, Lausanne University Hospital, Lausanne, Switzerland; 12 Lausanne University Hospital, Lausanne, Switzerland; 13 Gynecology and Obstetrics, Lausanne University Hospital, Lausanne, Switzerland

## Abstract

**Background:**

Hyperglycaemic disorders of pregnancy are associated with offspring cardiovascular alterations.

**Methods:**

MySweetHeart cohort study aimed to assess the effect of maternal gestational diabetes (GDM) on offsprings’ cardiovascular health. Newborns underwent clinical and echocardiographic examinations between 2016 and 2020.

**Results:**

Compared to mothers without GDM (*n* = 141), mothers with GDM (*n* = 123) were more likely to have had GDM in previous pregnancies and had higher weight, BMI, blood glucose, and HbA1c. Newborns of both groups showed similar clinical characteristics. Echocardiography was performed on the 3rd (interquartile range, IQR, 2nd–4th) day of life in 101 offsprings of mothers without and 116 offsprings of mothers with GDM. Left ventricular (LV) mass was similar. Children born to mothers with GDM had a thicker posterior LV wall (*z*-score +0.15, IQR –0.38/0.62, versus +0.47, IQR –0.11/+1.1, *p* = 0.004), a smaller end-systolic (1.3 mL, IQR 1.0–1.5 mL, versus 1.4 mL, IQR 1.2–1.8 mL, *p* = 0.044) but a similar end-diastolic LV volume. They also had shorter tricuspid valve flow duration and aortic valve ejection time, lower tricuspid E-wave and pulmonary valve velocities.

**Conclusions:**

Newborns of mothers with or without GDM had similar clinical characteristics and LV mass. However, some echocardiographic differences were detected, suggesting an altered myocardial physiology among infants of mothers with GDM.

**Registration:**

ClinicalTrials.gov (NCT02872974).

**Impact:**

- Hyperglycaemic disorders of pregnancy are known to be associated with offspring cardiovascular alterations.
- Clinical characteristics and estimated left ventricular (LV) mass were similar in children issued from mothers with and without gestational diabetes (GDM).
- Children born to mothers with GDM had a thicker posterior LV wall and a smaller end-systolic LV volume.
- Although LV mass is not different, myocardial physiology may be altered in these infants. Further studies should investigate the endothelial function of this population and the cardiovascular evolution of these children over time.

## Introduction

Arteriosclerosis is the leading cause of death worldwide.^1^ Besides traditional cardiovascular risk factors such as arterial hypertension, smoking, hyperlipidemia, diabetes mellitus, obesity, sedentary lifestyle and genetic predisposition, fetal programming and conditioning have been shown to play a relevant role, tracking into adulthood.^2–5^

Maternal hyperglycaemic disorders during pregnancy are associated with fetal cardiovascular alterations.^6^ In addition to an increased risk of congenital heart anomalies and septal hyperthrophy,^7^ typically described in the context of diabetes mellitus, more subtle changes can be found in the broader context of pregnancy hyperglycaemia. In fact, this latter might play a role in the fetal programming of obesity and metabolic disorders later in life.^6,8,9^

To investigate the possible role of gestational diabetes mellitus (GDM) on offspring’s cardiovascular health early in life, the MySweetHeart Cohort study aimed at assessing surrogate markers of cardiovascular health and disease during fetal life and shortly after birth.^6^ In the present contribution, we report the results relating to clinical and echocardiographic newborns’ characteristics.

## Methods

### Study design and study population

MySweetHeart Cohort is a study, whose protocol has been previously described.^6^ Briefly, all pregnant women attending the antenatal care or the GDM clinics at the Lausanne University Hospital (CHUV), Switzerland, or being followed by a gynecologist in private practice in the “Canton de Vaud” (Switzerland), were invited to participate. Eligible were pregnant women 18 or more years of age at 24–32 gestational weeks. Women not understanding either French or English, on strict bed rest, with pre-existing diabetes mellitus, or with severe mental disorders were excluded. Informed consent was sought and obtained by all participant mothers.

Diagnosis of GDM was made according to international recommendations.^6,10^ At each visit, data on maternal demographics, clinical and biochemical characteristics were collected, as described previously.^6^ Within 7 postnatal days, newborns were examined by an experienced pediatric cardiologist (blinded with respect to the maternal glycaemic status) by means of a thorough clinical assessment and a complete echocardiographic study.

### Echocardiography and measurements

Echocardiography was performed according to the recommendations of the American Society of Echocardiography^11^ on a Philips EPIQ 5 ultrasound system equipped with S8-3 and S12 MHz pediatric transducers. The following parameters were systematically assessed and transferred to a dedicated, anonymized database: (1) right (RA) and left atrial (LA) planimetered areas, measured from apical 4-chamber views;^11^ (2) Left ventricle (LV) size in both M-mode (short-axis) and 2D images, (3) LV function as estimated by means of fractional shortening (FS) in M-mode and ejection fraction (EF) according to the Simpson monoplane method; (4) flow measurements over atrioventricular and semilunar valves were taken with pulsed-wave, respectively continuous-wave Doppler, as appropriate; (5) diastolic function was assessed by means of mitral and tricuspid valves E-wave and A-wave measurements, E/A ratio and deceleration time, as well as aortic and pulmonary valve ejection times. Body surface area (BSA) was estimated by means of the Haycock method,^12^ left ventricular mass was estimated according to Devereux (LVM) and Reichek (LVM 2d) formulas,^13,14^ left ventricular mass index (LVMI) was estimated applying the de Simone correction to the Devereux formula.^15^

### Statistical analysis

Data were tested for normality graphically and by means of the D’Agostino–Pearson test. Since several variables were non-normally distributed, for consistency, all continuous variables are presented as median and interquartile range (IQR), respectively as box plots, and were compared by means of the non-parametric Mann–Whitney *U* test for independent samples. Proportions are presented as absolute number and percentage and variables were compared by means of Fisher’s exact test. Statistical significance was assigned at *p* < 0.05 (two-tailed). In a post-hoc analysis, we calculated the Spearman correlation coefficient *r* between maternal body mass index (BMI), maternal fasting plasma glucose, respectively, maternal HbA1c and several infants’ echocardiographic characteristics. Statistical analysis was performed with GraphPad Prism 8.0 (GraphPad Software, Inc., San Diego, California). Power analysis had previously indicated that a sample size of *n* = 80 per group would have been needed to detect a clinically significant difference in LVMI between the two groups and a conservative recruitment sample of *n* = 100 pregnant women per group was aimed for.^6^

## Results

### Characteristics of the study population

Between September 1, 2016, and October 24, 2020, 264 mothers with their newborn infants were included. Median maternal age was 33 (IQR 30–36) years, without significant differences between mothers with and without gestational diabetes. While no significant difference in language and education was detected, mothers with gestational diabetes were more likely to have smoked (*p* < 0.0001) or drunk (*p* = 0.0239) during pregnancy, to have already had gestational diabetes in previous pregnancies (*p* < 0.0001), to have a family history of diabetes mellitus (*p* = 0.0001), or to have a history of arterial hypertension (*p* = 0.0197). Interestingly, also a paternal family history of diabetes mellitus was more frequent (*p* = 0.0037) among children whose mothers had GDM (Table 1). Unsurprisingly, mothers with gestational diabetes were of higher weight (*p* < 0.0001) and BMI (*p* < 0.0001) and, as per definition, presented higher (*p* < 0.0001) blood glucose (both with void stomach and upon oral glucose provocation testing) and HbA1c (Table 1, *p* < 0.0001).

**Table 1:** Characteristics of study participants (mothers and fathers) with and without gestational diabetes.

	Without GDM	With GDM	*p* value
	(*N* = 141)	(*N* = 123)	
*Maternal demographic characteristics*			
Speaks French (y:n)	118:22	108:10	ns
Maternal education			ns
No scholarity	0	1	
Primary school	13	9	
Professional training	25	23	
Maturity	18	14	
University	81	52	
No answer	4	24	
General health: previous health problems (y/n)	63:68	49:38	ns
Previous GDM (y:n)	0:136	17:44	*p* < 0.0001
Family history of DM (maternal family) (y:n)	60:77	78:36	*p* = 0.0001
Family history of DM (paternal family) (y:n)	23:113	34:68	*p* = 0.0037
Father with DM (y:n)	1:133	3:104	ns
Maternal history of arterial HTN	0:135	3:48	0.0197
Maternal history of PCOS	0:135	1:46	ns
Maternal history of (previous) macrosomia	4:132	3:43	ns
Smoking (during pregnancy) (y:n)	5:135	22:93	*p* < 0.0001
Drinking during pregnancy	1:138	7:105	*p* = 0.0239
Recreational drugs during pregnancy	1:136	3:107	ns
*Maternal clinical characteristics*	(*N*)	(*N*)	
Age	33 [30–35]	33 [30–36]	ns
Parity (nulliparous: non-nulliparous)	78:62	58:60	ns
Body weight [kg]	59.6 [54.5–66.0]	66.5 [58.0–76.3]	*p* < 0.0001
Height [m]	1.65 [1.60–1.70]	1.63 [1.60–1.68]	ns
BMI [kg/m^2^]	21.8 [20.0–24.5]	24.8 [21.8–28.1]	*p* < 0.0001
Glucose void stomach [mmol/L]	4.5 [4.3–4.8]	5.1 [4.8–5.4]	*p* < 0.0001
Glucose 1 h post-challenge [mmol/L]	6.9 [6.0–8.3]	10.2 [8.4–11.0]	*p* < 0.0001
Glucose 2 h post-challenge [mmol/L]	6.0 [4.2–6.8]	8.2 [6.8–9.2]	*p* < 0.0001
HbA1c [%]	4.8 [4.7–5.1]	5.2 [5.0–5.4]	*p* < 0.0001

*BMI* body mass index, *DM* diabetes mellitus, *GDM* gestational diabetes mellitus, *HbA1c* glycated hemoglobin, *HTN* hypertension, *n* no, *PCOS* polycystic ovary syndrome, *y* yes.

Newborns issued from mothers with and without gestational diabetes did not show any significant difference in their baseline clinical characteristics (Table 2).

**Table 2:** Clinical characteristics of participant newborns issued from mothers with and without gestational diabetes.

	Without GDM	With GDM	*p* value
	(*N* = 141)	(*N* = 123)	
*Infant clinical characteristics*			
Delivery mode			ns
Vaginal	104	78	
Cesarean section	28	36	
Unknown	4	3	
Age at echocardiography [days]	3 [2–5]	3 [2–4]	ns
Weight at echocardiography [kg]	3.2 [2.9–3.6]	3.3 [3.1–3.6]	ns
Height at echocardiography [cm]	50 [48–51]	50 [48–51]	ns
BSA at echocardiography [m^2^]	0.20 [0.20–0.21]	0.20 [0.19–0.21]	ns
Sex (M:F)	73:68	65:58	ns
SBP [mmHg]	78 [72–84]	77 [71–83]	ns
DBP [mmHg]	46 [40–52]	47 [41–55]	ns
Heart rate [/min]	126 [112–144]	122 [108–136]	ns

*BSA* body surface area, *DBP* diastolic blood pressure, *F* female, *M* male, *SBP* systolic blood pressure.

### Echocardiographic measurements at birth

Echocardiographic measurements were available for 217 (82%) out of the 264 included children, of which 101 were among children born to mothers without and 116 among children born to mothers with GDM (Table 3), and were performed in median on the 3rd (IQR 2nd–4th) day of life. Both M-mode measurements and LVM estimations were available for at least 202 (93%) children, while a complete echocardiographic set of measures was obtained in at least 185 (85%) participants.

**Table 3:** Echocardiographic characteristics of participant newborns issued from mothers with and without gestational diabetes.

	Without GDM	With GDM	*p* value
	*n* = 101	*n* = 116	
Anatomy abnormal			ns
PFO/ASD	1	3	
PDA	1	4	
VSD	0	5	
SVC abnormality	0	1	
LV-FS [%]	38 [33–42]	38 [33–43]	ns
LV-EF (Simpson monoplan) [%]	65 [59–72]	68 [62–73]	ns
TAPSE [mm]	9.0 [8.1–10.0]	8.9 [8.0–9.8]	ns
IVS [mm]	3.5 [3.2–4.0]	3.6 [3.1–4.1]	ns
IVS [*z*-score]	−0.27 [−0.64/+0.15]	−0.10 [−0.73/+0.48]	ns
LVEDd [mm]	18 [16–19]	18 [17–19]	ns
LVEDd [*z*-score]	−0.40 [−0.72/+0.01]	−0.44 [−0.92/−0.14]	ns
LVESd [mm]	11 [9.8–12]	11 [9.7–12]	ns
LVESd [*z*-score]	−0.31 [−0.63/+0.14]	−0.38 [−0.90/+0.11]	ns
PWd [mm]	3.1 [2.7–3.5]	3.2 [2.9–3.7]	ns
PWd [*z*-score]	0.15 [−0.38/+0.62]	0.47 [−0.11/+1.1]	*p* = 0.0041
LVM 2D (Reichek) [g]	7.2 [6.5–8.3]	7.6 [6.6–8.5]	ns
LVM (Devereux) [g]	7.9 [6.6–9.0]	7.8 [6.7–9.3]	ns
LVMI (de Simone G) [g/m^2.7^]	51.6 [46.1–59.5]	51.0 [45.7–59.6]	ns
RA area [cm^2^]	2.4 [1.9–2.5]	2.1 [1.9–2.5]	ns
LA area [cm^2^]	1.8 [1.6–2.2]	2.0 [1.7–2.2]	ns
LV ED-Vol [mL]	4.4 [3.5–5.3]	4.0 [3.4–4.8]	ns
LV ES-Vol [mL]	1.4 [1.2–1.8]	1.3 [1.0–1.5]	*p* = 0.0437
Vmax AoV [cm/s]	81 [72–93]	83 [72–93]	ns
Ejection time AoV [s]	0.20 [0.19–0.21]	0.20 [0.18–0.21]	*p* = 0.0282
Vmax PulmV [cm/s]	88 [75–101]	82 [71–90]	*p* = 0.015
Ejection time PulmV [s]	0.22 [0.20–0.24]	0.22 [0.20–0.24]	ns
MV E [cm/s]	55 [46–64]	55 [47–63]	ns
MV A [cm/s]	52 [44–61]	52 [44–60]	ns
MV E/A ratio	1.00 [0.84–1.26]	1.08 [0.88–1.22]	ns
MV flow duration [s]	0.25 [0.24–0.27]	0.25 [0.23–0.27]	ns
MV Deceleration time [s]	0.11 [0.09–0.13]	0.11 [0.09–0.13]	ns
TV E [cm/s]	50 [43–59]	46 [37–55]	*p* = 0.0258
TV A [cm/s]	57 [51–64]	55 [49–64]	ns
TV E/A ratio	0.86 [0.72–1.04]	0.80 [0.69–1.05]	ns
TV flow duration [s]	0.26 [0.24–0.29]	0.26 [0.24–0.27]	*p* = 0.0486
TV Deceleration time [s]	0.12 [0.09–0.15]	0.11 [0.09–0.14]	ns

*AoV* aortic valve, *ASD* atrial septal defect, *BSA* body surface area, *GDM* gestational diabetes mellitus, *LA* left atrium, *LV* left ventricle, *LV-EF* left ventricle ejection fraction, *LV-FS* left ventricle fractional shortening, *LVM* left ventricular mass, *LVMI* left ventricular mass index, *MV* mitral valve, *PFO* patent foramen ovale, *PulmV* pulmonary valve, *RA* right atrium, *RV* right ventricle, *SVC* superior vena cava, *TAPSE* tricuspid annular plane systolic excursion, *TV mitral valve, Vmax* instantaneous maximal velocity, *VSD* ventricular septal defect.

**Fig. 1:**
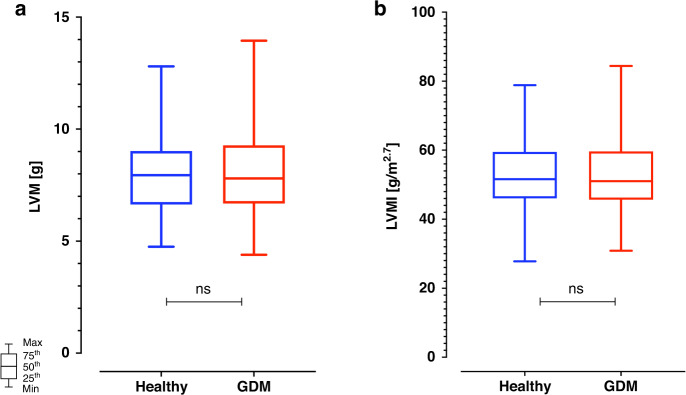
Box plots of the estimated left ventricular mass and mass index. Box plots of the estimated left ventricular mass (LVM, **a**) for children born to mothers with and without gestational diabetes, as calculated by the Devereux formula. No significant difference between the two groups was detected. Similarly (**b**), no significant difference was detected while comparing the left ventricular mass index (LVMI), as calculated by the de Simone formula.

As per the primary outcome, there was no significant difference in estimated LVM in children born to mothers with and without gestational diabetes, irrespective of which formula was used to echocardiographically estimate LVM (Table 3) and of whether the absolute mass or the indexed value was considered (Fig. 1).

**Fig. 2:**
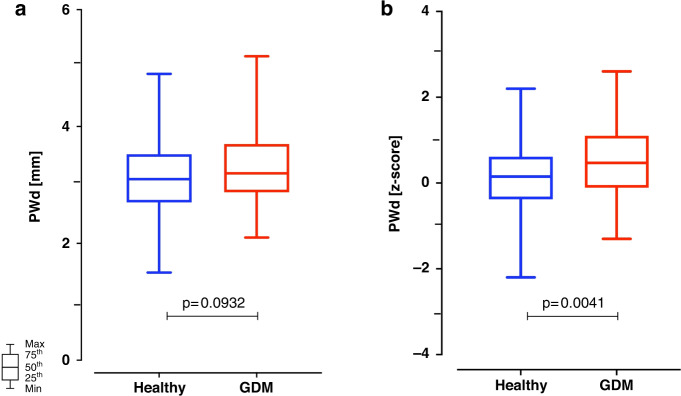
Box plots of posterior left ventricular wall thickness (PWd). The posterior left ventricular wall thickness (PWd) was similar in children born to mothers with (3.2, IQR 2.9–3.7 mm) or without (3.1, IQR 2.7–3.5 mm; *p* = 0.093) gestational diabetes when assessed as absolute dimension (**a**). However, it was slightly but significantly higher in children born to mothers with gestational diabetes (+0.47, IQR –0.11 to +1.1) than without (+0.15, IQR 0.38 to +0.62; *p* = 0.004) when normalized and assessed as *z*-scores (**b**).

**Fig. 3:**
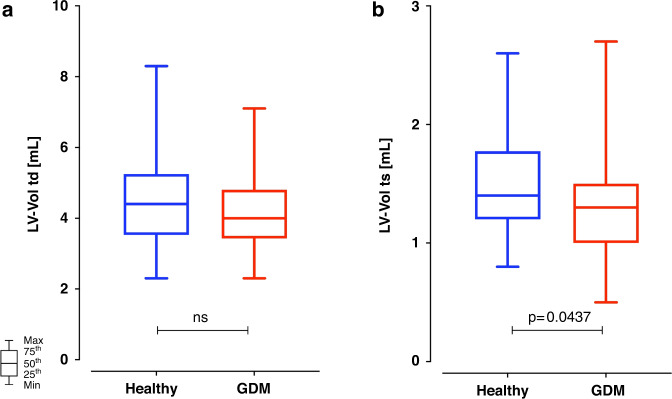
Box plots of left ventricular end-diastolic (LV-Vol td) and systolic (LV-Vol ts) volumes. The left ventricular end-diastolic volume (LV-Vol td, **a**) was similar in children born to mothers with (4.0, IQR 3.4–4.8 mL) or without (4.4, IQR 3.5–5.3 mL, *p* = 0.113) gestational diabetes. However, the systolic left ventricular volume (LV-Vol ts, **b**) was slightly but significantly smaller in children born to mothers with gestational diabetes (1.3, IQR 1.0–1.5 mL) than without (1.4, IQR 1.2–1.8 mL; *p* = 0.044).

Although most echocardiographic parameters were similar while comparing the two groups, there were a few significant differences. Children born to mothers with gestational diabetes had a thicker posterior LV wall, as assessed by *z*-scores (+0.15, IQR 0.38–0.62, versus +0.47, IQR –0.11/+1.1, *p* = 0.0041), while the comparison was not significant when the LV posterior wall was simply assessed as an absolute dimension (Fig. 2). While the end-diastolic volume was not significantly different, the end-systolic LV volume was (Fig. 3), with control children presenting a slightly, but significantly higher end-systolic LV volume (1.4, IQR 1.2–1.8 mL) than children issued from a pregnancy characterized by gestational diabetes (1.3, IQR 1.0–1.5 mL, *p* = 0.0437). Aortic valve ejection time and pulmonary valve maximal velocity were minimally, but significantly, smaller among children born to mothers with gestational diabetes than without (Table 3). Both tricuspid valve E-wave and tricuspid valve flow duration were minimally but significantly higher, respectively longer, among controls than among children born to mothers with gestational diabetes (Table 3).

Maternal BMI was weakly but significantly associated with LV-EF (*r* = 0.1429, 95% confidence interval –0.001701 to 0.2817; *p* = 0.0462), LVM as calculated with the Reichek formula (*r* = 0.1785, 95% confidence interval 0.03670–0.3133, *p* = 0.0114) and the mitral valve E/A ratio (*r* = -0.1752, 95% confidence interval –0.3119 to –0.03142; *p* = 0.0143). No significant correlation between BMI and the other considered echocardiographic parameters was detected. Maternal fasting plasma glucose level was not significantly associated with any echocardiographic parameter. Maternal HbA1c was significantly associated with LV posterior wall diameter (*r* = 0.1681, 95% confidence interval 0.01928–0.3096, *p* = 0.0229) and LV posterior wall *z*-score (0.2387, 95% confidence interval 0.08559–0.3808, *p* = 0.0019), without any further significant correlation between HbA1c and the remaining echocardiographic parameters.

## Discussion

The present study was able to analyze 123 mother-infant diads with gestational diabetes compared to 141 diads without, and delivered three main results. (1) Although clinical characteristics of mothers were different between groups, those of infants were comparable. (2) Contrary to what we expected, the estimated left ventricular mass and mass index were not different among children issued from pregnancies with or without gestational diabetes. (3) However, some trivial but significant differences were found in a few echocardiographic parameters: infants born to mothers with gestational diabetes had a thicker posterior LV wall, smaller end-systolic left ventricular volume, shorter tricuspid valve flow duration and aortic valve ejection time, lower tricuspid E-wave and pulmonary valve maximal velocities.

These results represent novel knowledge. The differences being minimal, it is difficult to imagine an immediate clinical impact of this study. However, this data bears significant scientific interest and suggests that, although morphological differences are limited, distinctive functional variations can be detected in children born to mothers with gestational diabetes. Considering the important burden of cardiovascular disease over a lifetime course, the potential impact of better understanding these phenomena is relevant.

While interpreting the results, six considerations deserve discussion. First, the differences in baseline clinical and biochemical characteristics of mothers with and without gestational diabetes are all but surprising, and partly simply reflect the definition of this condition. Second, the fact that no significant clinical differences were detected among infants, including similar weight, height, BSA, and blood pressure, might apparently be surprising. However, this study was observational. This implies that mothers with gestational diabetes were managed as per clinical routine and all efforts were made to achieve normal glycemic control throughout the pregnancy. Third, this same consideration might explain the absence of significant difference in estimated left ventricular mass and mass index while comparing infants of the two groups (i.e., the primary outcome). Fourth, both the higher posterior left ventricular wall and the smaller end-systolic left ventricular volume in infants issued from pregnancies with gestational diabetes might suggest some minimal difference towards increased myocardial substance (even if not already reflected in significant estimated mass increase). It is tempting to assume that the careful clinical management was able to avoid macrosomia and left ventricular hypertrophy, but not to abolish any more subtle difference in myocardial structure and thickness. Fifth, the shorter tricuspid valve flow duration and aortic valve ejection time, as well as the lower tricuspid E-wave and pulmonary valve maximal velocities in infants born to mothers with gestational diabetes are tricky to interpret. A shorter tricuspid valve flow duration and lower E-wave velocity might suggest a more pronounced diastolic filling impairment of the right ventricle in infants born to mothers with gestational diabetes, but it is surprising that this difference was not detected while analyzing the mitral valve inflow into the LV. Furthermore, both tricuspid wave E/A ratio and deceleration time, which are further right ventricular diastolic function parameters, were not different among groups. The shorter aortic valve ejection time might suggest an increased systemic vascular resistance, but this was unfortunately not measured, so we cannot either prove or reject this hypothesis. The lower pulmonary valve maximal velocity in infants issued from a pregnancy with gestational diabetes remains without sensible physiological explanation. Finally, the post-hoc correlation analysis, although just explorative, confirmed a positive correlation between maternal BMI and both LVM (as assessed by the Reichek formula) and the LV-EF, as well as an impaired LV relaxation, as expressed by a negative correlation with the mitral valve E/A ratio. Interestingly, while maternal fasting plasma glucose was not correlated with any infant echocardiographic parameter, maternal HbA1c was positively correlated with LV posterior wall diameter. In summary, although explorative and not adequately powered, correlation analysis was in line with the global interpretation of the main results: some weak association with a limited set of echocardiographic parameters.

This study has some strengths and several limitations. First, for a pediatric study, we were able to collect data from a pretty numerous population, meeting the pre-determined sample size. Second, the involvement of both private gynecological practices and a University hospital allows extrapolating to both clinical contexts. At the same time, the centralized pediatric cardiologic assessment ensured consistency in clinical evaluation, echocardiographic equipment, and measurements. Third, a numerous set of echocardiographic measurements were performed, allowing to investigate several aspects of the myocardium. However, different limitations must be acknowledged. First, since mothers with gestational diabetes were managed according to routine care and efforts were put in place to achieve a normal glycemic status, the effect size was very small, and therefore differences were difficult to detect. Second, intrinsic to echocardiography in the neonatal population, the measured structures are small and echocardiographic measurements therefore prone to errors. Even if these are clinically irrelevant, they may impact scientific studies, especially when using raw data to perform calculations (e.g., mass estimations). Third, no data on endothelial function was gathered. Unfortunately, owing to organizational, ethical and financial constraints, we were not able to collect pulse wave velocity and biochemical markers of endothelial function. According to current research, these might however be helpful in investigating such populations.^16–21^ In fact, significantly increased E-selectin, vascular adhesion molecule 1 and waist circumference have been detected in offspring of diabetic pregnancies.^22^ Nevertheless, in the context of the MySweetHeart Cohort study, we also measured intima media thickness, a marker of endothelial structural changes.^23^ These results have recently been reported.^24^ Fourth, the effects of maternal glycaemia are far beyond myocardial structure. In fact, they may impact fetal brain activity and appetite regulation, programming future nutritional behavior.^25^ These aspects were not investigated in the current study. Fifth, no biochemical data (e.g., plasma glucose and prevalence of hypoglycaemia, growth hormone, insulin-like growth factor 1, Ca^2+^, Mg^2+^, etc.) of included newborns were collected. Finally, this was a cross-sectional analysis, and no follow-up measurements are currently available. In fact, some consequences of the exposure to maternal diabetes mellitus or gestational diabetes might manifest (only) later in life.^26–29^ For example, according to a recent meta-analysis, children born to mothers with gestational diabetes had increased systolic blood pressure (measured at the age of 3–16 years), BMI (measured at the age of 3–15 years) and serum glucose (measured at 8–27 years of age),^30^ so that medium and long-term follow-up studies are warranted, even in the absence of significant differences short after birth. Notably, in a study among 99 offspring of diabetic mothers and 422 controls, children born to mothers with gestational diabetes showed a decreased adiponectin and an increased leptin at 6–13 years of age,^22^ while in a similar study no difference was detected at 1 year of age.^31^

In conclusion, while comparing newborns issued from pregnancies with and without gestational diabetes, this study showed no difference either in infant baseline clinical characteristics or in their echocardiographically estimated LVM. However, some minor echocardiographic differences were detected, suggesting that myocardial physiology may be altered in these infants. Further studies should investigate the endothelial function of this population and the cardiovascular evolution of these children over time.

## Data Availability

The data that support the findings of this study are available from the corresponding author upon reasonable request.

## References

[1] GBD 2016 Causes of Death Collaborators. (2017). Global, regional, and national age-sex specific mortality for 264 causes of death, 1980-2016: a systematic analysis for the Global Burden of Disease Study 2016. Lancet.

[2] Charakida M, Deanfield JE, Halcox JP (2007). Childhood origins of arterial disease. Curr. Opin. Pediatr..

[3] Barker DJ, Osmond C, Golding J, Kuh D, Wadsworth ME (1989). Growth in utero, blood pressure in childhood and adult life, and mortality from cardiovascular disease. BMJ.

[4] Barker DJ, Eriksson JG, Forsén T, Osmond C (2002). Fetal origins of adult disease: strength of effects and biological basis. Int J. Epidemiol..

[5] Franks PW (2010). Childhood obesity, other cardiovascular risk factors, and premature death. N. Engl. J. Med..

[6] Di Bernardo S (2017). Assessing the consequences of gestational diabetes mellitus on offspring’s cardiovascular health: MySweetHeart Cohort study protocol, Switzerland. BMJ Open.

[7] Ullmo S (2007). Pathologic ventricular hypertrophy in the offspring of diabetic mothers: a retrospective study. Eur. Heart J..

[8] Nolan CJ, Damm P, Prentki M (2011). Type 2 diabetes across generations: from pathophysiology to prevention and management. Lancet.

[9] Hanson MA, Gluckman PD (2014). Early developmental conditioning of later health and disease: physiology or pathophysiology?. Physiol. Rev..

[10] Metzger BE (2010). International association of diabetes and pregnancy study groups recommendations on the diagnosis and classification of hyperglycemia in pregnancy. Diabetes Care.

[11] Lopez L (2010). Recommendations for quantification methods during the performance of a pediatric echocardiogram: a report from the Pediatric Measurements Writing Group of the American Society of Echocardiography Pediatric and Congenital Heart Disease Council. J. Am. Soc. Echocardiogr..

[12] Haycock GB, Schwartz GJ, Wisotsky DH (1978). Geometric method for measuring body surface area: a height-weight formula validated in infants, children, and adults. J. Pediatr..

[13] Devereux RB (1986). Echocardiographic assessment of left ventricular hypertrophy: comparison to necropsy findings. Am. J. Cardiol..

[14] Reichek N, Helak J, Plappert T, Sutton MS, Weber KT (1983). Anatomic validation of left ventricular mass estimates from clinical two-dimensional echocardiography: initial results. Circulation.

[15] de Simone G (1992). Left ventricular mass and body size in normotensive children and adults: assessment of allometric relations and impact of overweight. J. Am. Coll. Cardiol..

[16] Laurent S (2006). Expert consensus document on arterial stiffness: methodological issues and clinical applications. Eur. Heart J..

[17] Im JA, Lee JW, Shim JY, Lee HR, Lee DC (2007). Association between brachial-ankle pulse wave velocity and cardiovascular risk factors in healthy adolescents. J. Pediatr..

[18] Lehmann ED (1999). Clinical value of aortic pulse-wave velocity measurement. Lancet.

[19] Townsend RR (2015). Recommendations for improving and standardizing vascular research on arterial stiffness: a scientific statement from the American Heart Association. Hypertension.

[20] Fenton M (2016). Inflammatory cytokines, endothelial function, and chronic allograft vasculopathy in children: an investigation of the donor and recipient vasculature after heart transplantation. Am. J. Transplant..

[21] Močnik M, Marčun Varda N (2022). Current knowledge of selected cardiovascular biomarkers in pediatrics: kidney injury Molecule-1, Salusin-α and -β, Uromodulin, and Adropin. Child. (Basel).

[22] West NA, Crume TL, Maligie MA, Dabelea D (2011). Cardiovascular risk factors in children exposed to maternal diabetes in utero. Diabetologia.

[23] Epure AM (2020). Risk factors during first 1,000 days of life for carotid intima-media thickness in infants, children, and adolescents: a systematic review with meta-analyses. PLoS Med.

[24] Epure AM (2022). Gestational diabetes mellitus and offspring’s carotid intima-media thickness at birth: MySweetHeart Cohort study. BMJ Open.

[25] McIntyre D (2018). FIGO analysis of research priorities in hyperglycemia in pregnancy. Diabetes Res Clin. Pract..

[26] Crume TL (2011). The impact of in utero exposure to diabetes on childhood body mass index growth trajectories: the EPOCH study. J. Pediatr..

[27] Lee H, Jang HC, Park HK, Cho NH (2007). Early manifestation of cardiovascular disease risk factors in offspring of mothers with previous history of gestational diabetes mellitus. Diabetes Res Clin. Pract..

[28] Wahab RJ (2020). Maternal glucose concentrations in early pregnancy and cardiometabolic risk factors in childhood. Obesity (Silver Spring)..

[29] Mitanchez D, Burguet A, Simeoni U (2014). Infants born to mothers with gestational diabetes mellitus: mild neonatal effects, a long-term threat to global health. J. Pediatr..

[30] Pathirana MM, Lassi ZS, Roberts CT, Andraweera PH (2020). Cardiovascular risk factors in offspring exposed to gestational diabetes mellitus in utero: systematic review and meta-analysis. J. Dev. Orig. Health Dis..

[31] Retnakaran R (2013). Effect of maternal gestational diabetes on the cardiovascular risk factor profile of infants at 1 year of age. Nutr. Metab. Cardiovasc Dis..

